# Better Response to Influenza Virus Vaccination in Physically Trained Older Adults Is Associated With Reductions of Cytomegalovirus-Specific Immunoglobulins as Well as Improvements in the Inflammatory and CD8^+^ T-Cell Profiles

**DOI:** 10.3389/fimmu.2021.713763

**Published:** 2021-10-12

**Authors:** Eduardo S. Felismino, Juliana M. B. Santos, Marcelo Rossi, Carlos A. F. Santos, Edison L. Durigon, Danielle B. L. Oliveira, Luciano M. Thomazelli, Fernanda R. Monteiro, Adriane Sperandio, Juliana S. Apostólico, Carolina N. França, Jonatas B. Amaral, Gislene R. Amirato, Rodolfo P. Vieira, Mauro Vaisberg, André L. L. Bachi

**Affiliations:** ^1^ Post-Graduation Program in Health Science, University of Santo Amaro, São Paulo, Brazil; ^2^ Post-Graduation Program in Sciences of Human Movement and Rehabilitation, Federal University of São Paulo, Santos, Brazil; ^3^ Ear, Nose and Throat (ENT) Lab, Department of Otorhinolaryngology, Federal University of São Paulo, São Paulo, Brazil; ^4^ Department of Medicine, Geriatry, Paulista School of Medicine (EPM), São Paulo, Brazil; ^5^ Laboratory of Clinical and Molecular Virology, Department of Microbiology, Institute of Biomedical Science of University of São Paulo, São Paulo, Brazil; ^6^ Scientific Platform Pasteur–University of São Paulo, São Paulo, Brazil; ^7^ Hospital Israelita Albert Einstein, São Paulo, Brazil; ^8^ Method Faculty of São Paulo, São Paulo, Brazil; ^9^ Department of Microbiology, Immunology and Parasitology, Federal University of São Paulo, São Paulo, Brazil; ^10^ Post-Graduation Program in Bioengineering, Universidade Brasil, São Paulo, Brazil; ^11^ Brazilian Institute of Teaching and Research in Pulmonary and Exercise Immunology (IBEPIPE), Sao Jose dos Campos, Brazil

**Keywords:** cytomegalovirus, influenza virus vaccine, cytokines, immunoglobulin, exercise training, elderly

## Abstract

Chronic cytomegalovirus (CMV) infection is a trigger factor for the development of immunosenescence and negatively impacts the immune response to influenza virus vaccination (IVV) in older adults. However, the role of physical exercise training in this context is unknown. Thus, the aim of this study was to investigate whether the regular practice of combined exercise training can improve the specific antibody response to IVV in CMV-seropositive older adults. Eighty older adults were distributed into two groups—non-practitioners (NP, *n* = 31, age = 74.06 ± 6.4 years) and practitioners of combined exercise training (CET, *n* = 49, age = 71.7 ± 5.8 years)—for at least 12 months. Both volunteer groups were submitted to IVV and blood samples were collected before (pre) and 30 days after (post) the vaccination. Concerning the specific antibody response to IVV, higher serum levels of specific immunoglobulin A (IgA) were found in the CET group post- than pre-vaccination (*p* < 0.01), whereas higher levels of specific immunoglobulin M (IgM) were observed both in the NP (*p* < 0.05) and CET (*p* < 0.001) groups post-vaccination as compared to the pre-vaccination values. Serum levels of specific immunoglobulin G (IgG) for IVV and CMV, as well as interleukin 6 (IL-6) and IL-10, were similar between the time points evaluated. However, the IL-10/IL-6 ratio post-vaccination was higher (*p* < 0.05) in the CET group than that before vaccination. Negative correlations were observed between the specific IgG levels for IVV and CMV only in the CET group, both pre- and post-vaccination. In addition, negative correlations were found between IL-10 and specific IgG for CMV in all volunteer groups pre- and post-vaccination, whereas a positive correlation between IL-10 and specific-IgG for IVV pre- and post-vaccination was observed in the CET group. In addition, with the hemagglutination inhibition (HAI) assay, it was found that 32.2% of the NP group and 32.6% of the CET group were responders to IVV and displayed reductions in the CMV serostatus (*p* < 0.05 and *p* < 0.001, respectively) and increases in naive and effector CD8^+^ T cells post-vaccination (*p* < 0.01). However, only the responders from the CET group showed significant reductions in the ratio of effector to naive CD8^+^ T cells (*p* < 0.05) and increased IL-10 levels post-vaccination (*p* < 0.001). In summary, this study demonstrates that the improvement in the response to IVV in CMV-seropositive older adults was related to an anti-inflammatory status and enhancement of naive CD8^+^ T cells, particularly associated with regular practice of CET.

## Introduction

Among some of the issues concerning the aging process, the reduction of immunological activities, a phenomenon named immunosenescence, is considered a corollary factor that leads the older population to present decreased responses to vaccination. Even though vaccination is a safe intervention that, in a general way, can elicit an immunological protective response from infants to older people, the literature highlights the immunogenicity in vaccination, especially to the influenza virus vaccination (IVV), which is influenced negatively by age since individuals aged 50–54 years show vaccine effectiveness of around 52% *vs*. only 40% in older adults aged ≥65 years (1, 2).

Respiratory viruses are closely associated with severe diseases in the older population; particularly, the influenza virus is the main cause of the increased age-related hospitalization and death. In this sense, it is widely accepted that the annual vaccination campaign for the influenza virus, mainly for the older population, can avoid, or at least minimize, the severity of this infection (1).

As cited above, immunosenescence compromises the vaccine response not only due to the suppression of naive T cells in association with the accumulation of effector and memory T cells but also as a consequence of the development of the inflammaging phenomenon (3, 4). According to the literature, inflammaging is a chronic, systemic, sterile, low-grade age-related inflammation in which the levels of pro-inflammatory cytokines, such as interleukin 6 (IL-6), are increased, in contrast to the reduction of anti-inflammatory cytokines such as IL-10 (5). It is of utmost importance to point out that the reactivation of infection by cytomegalovirus, a herpes virus, can be involved in the triggers of inflammaging.

Cytomegalovirus (CMV) infection often occurs during childhood and can elicit a strong immune response. Unfortunately, this immune response is not able to completely eliminate the virus, which leads to this infection becoming latent, persisting during life (6, 7). In this respect, CMV uses myeloid cells as its main reservoir and the host’s inflammatory response to perpetuate its life cycle, which can also prevent its elimination. It is paramount to highlight that pro-inflammatory cytokines can trigger reactivation of the virus from its latency state to active viral replication. Thus, repeated cycles of reactivation and replication can amplify the systemic pro-inflammatory status, especially in older adults (8–10). Interestingly, it has been demonstrated that CMV antibody titers are positively correlated with systemic pro-inflammatory cytokines and present a negative correlation with the antibody response to IVV, preferentially in older adults (11, 12).

Beyond this prominent association between CMV infection and humoral response and inflammatory status, alterations in cellular immunity, particularly a reduction in the frequency of CD8^+^ T cells, are one of the main hallmarks not only of aging but also of CMV infection. According to the literature, individuals with latent CMV infection frequently show a remarkable reduction in the frequency of naive T cells, particularly in CD8^+^ T cells in comparison to CD4^+^ T cells, when compared with individuals without CMV infection, and regardless of age. In contrast to these reductions in naive CD8^+^ T cells, CMV-seropositive individuals present elevations in the frequency of effector CD8^+^ T cells. Therefore, due to this feature being closely associated with immunosenescence and CMV-latency, it can have an impact on the reduction of the age-related response in influenza virus infection and its vaccination (13–20).

Despite immunosenescence and CMV infection being two factors that can deeply compromise the vaccination response, there is consensus that the regular practice of physical exercise is able to not only enhance the immune response but also regulate the systemic inflammatory status, which leads to an increase in immunogenicity for vaccination, including in older people (21). Corroborating the literature, our group has reported that older adults who practice regular exercise training present improvements in the cellular and humoral immune responses to IVV when compared not only to older non-practitioners of physical exercise training but also to those with a sedentary lifestyle (4, 22, 23).

Thus, the aim of this study was to investigate whether the regular practice of combined exercise training can improve specific antibody response to IVV in CMV-seropositive older adults.

## Material and Methods

### Subjects and Study Design

**Figure 1:**
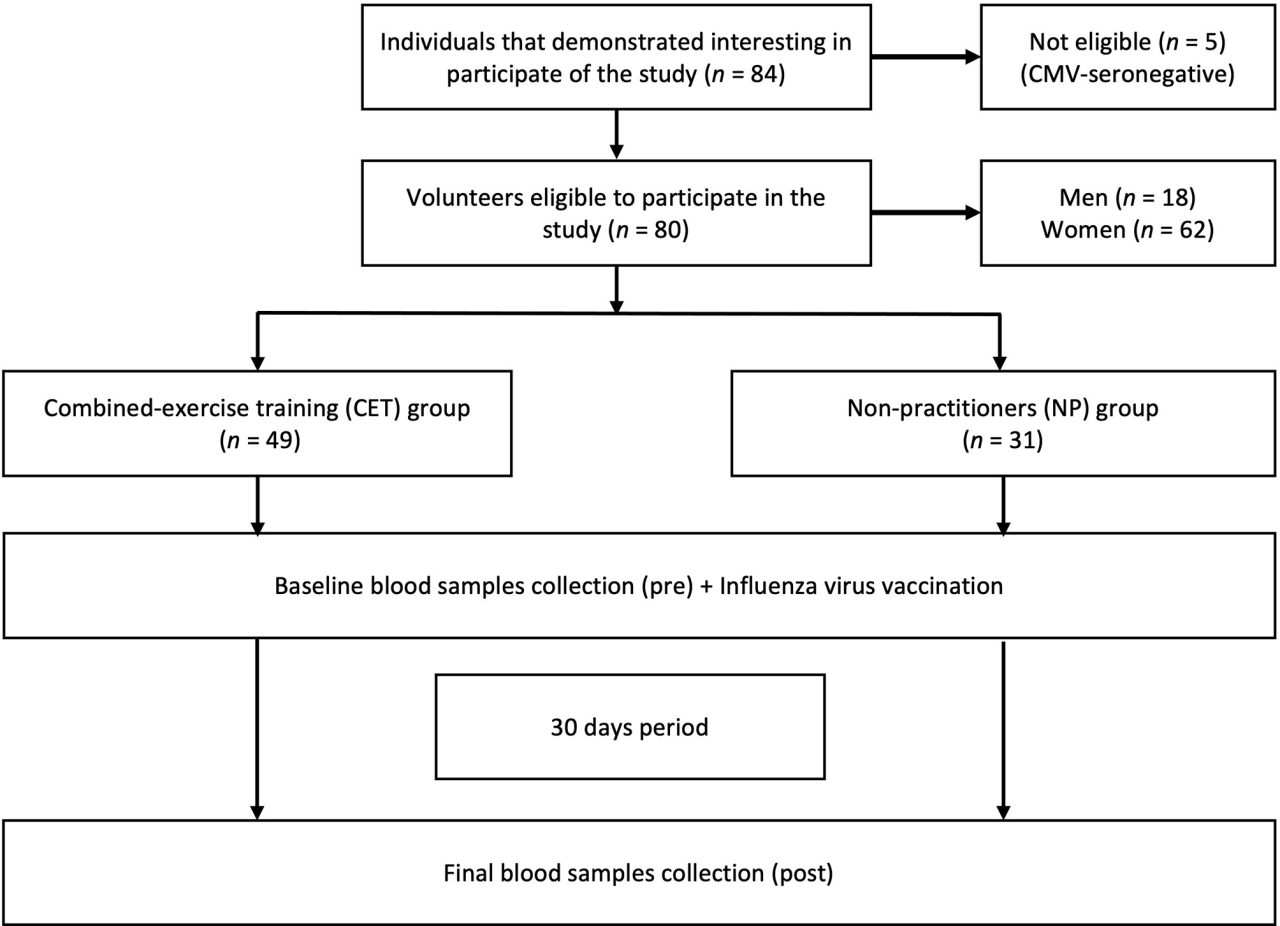
Flow diagram and experimental design.

As shown in the flow diagram (**Figure 1**), 80 older adults (aged 60–85 years), both women (*n* = 62) and men (*n* = 18), were enrolled in this study. The selection and recruitment were carried out by the coordinator of the Primary Health Care Program for Older People from the Geriatrics and Gerontology Discipline of the Federal University of São Paulo (UNIFESP). Thereafter, the recruited volunteers received all pieces of information concerning the study before giving their written informed consent. It is noteworthy clarifying that the study was approved both by the National Research Ethics Committee (no. CAEE:218170619.3.0.000.5505) and by the Ethics Committee of the Federal University of São Paulo (approval no. 3.623.247). In addition, all experiments were performed in accordance with the Declaration of Helsinki (24).

According to **Figure 1**, by the selection and recruitment process, the volunteers in this study were allocated into two groups: practitioners of combined exercise training (CET, *n* = 49) and non-practitioners (NP, *n* = 31). In this respect, it is worth highlighting that, during the study period, all volunteers maintained their daily routine. Furthermore, blood samples were collected at two different time points: before (pre) and 30 days after (post) the IVV (**Figure 1**).

### Evaluations of the Anthropometric, Nutritional, and Clinical Characteristics and the Physical Activity Levels

Anthropometric data [weight, height, and body mass index (BMI)] and body composition by bioimpedance (BIOSCAN 920-2-S, Maltron International Limited, Rayleigh, UK) were evaluated. Data related to daily nutritional consumption (macronutrients and micronutrients) were assessed using the Food Frequency Questionnaire. Also, the volunteers showed a diet with more than 1.75 g of protein ingestion per kilogram of body mass, and none of them were taking multivitamin/antioxidant supplements (exclusion criterion 1).

Regarding the clinical and physical examinations, it is important to mention that the same geriatric physician performed these exams. It was reported that none of the volunteers presented neoplasia, HIV, and/or other chronic infections, neurological, liver, and/or renal diseases, type I diabetes, and diseases that precluded the performance of the physical exercises determined in this study protocol (exclusion criterion 2). In addition, they did not use anti-inflammatory drugs in the last 2 months (exclusion criterion 3).

The International Physical Activity Questionnaire (IPAQ), which is validated for the Brazilian population (25, 26), was used to assess data on the daily physical activity levels of the volunteers. All of them presented more than 150 min/week of physical activity (exclusion criterion 4).

### Influenza Virus Vaccination

All participants in this study were submitted to the seasonal IVV in 2019. The trivalent vaccine was composed of two influenza A virus types [A/Michigan/45/2015 (H1N1) and A/Switzerland/8060/2017(H3N2)] and one influenza B virus type (B/Colorado/06/2017). Importantly, all the older participants in this study were submitted to a trivalent inactivated vaccine (TIV) dose of 15 μg of hemagglutinin (HA)/virus, which was offered to the Brazilian population during the Flu Campaign in 2019 by the national government.

### CET Program

Briefly, the CET (a combination of resistance and aerobic exercises) consisted of a program of 60–75 min of exercise training per session, performed three times per week, on alternate days, for 30 days. It is worthy citing that the volunteers in the CET group performed this program for at least 12 months, and the same physical education professional was responsible for supervising them during the study period. More details concerning the CET program performed by these volunteers can be found in Bachi et al. (4, 27).

### Collection of Blood Samples

Blood samples were collected in tubes without any anticoagulant agent at two different time points: prior (pre) and 30 days after (post) IVV. Baseline blood sampling was done in a general way, 30–45 min before the IVV for both groups of volunteers. Serum aliquots were obtained after blood coagulation in the tube and centrifugation (300 × *g*, 10 min at 4°C). These were used to assess the responses of specific antibodies [immunoglobulin A (IgA), IgM, and IgG] to IVV and specific IgG to CMV, as well as both pro-inflammatory (IL-6) and anti-inflammatory (IL-10) cytokines. It is important to clarify that those in the CET group were instructed to perform the last exercise training session 24 h before collection of the blood samples.

### Determination of Specific Antibody Response to IVV and CMV Serostatus

CMV serostatus was evaluated by the determination of the serum level of specific IgG for CMV using a commercial ELISA test kit (BioClin, MG, Brazil). The CMV seropositivity of the older adults was defined as an IgG concentration ≥1.32 IU/ml, in accordance with the manufacturer’s instructions. Based on this, five CMV-seronegative older adults (two from NP group and three from the CET group) were excluded from the study, as shown in the flow diagram (**Figure 1**).

For the evaluation of the serum levels of specific IgA, IgM, and IgG for the IVV, an “in-house” ELISA test was carried out following the procedure formerly presented by our group (4). Briefly, serum samples were diluted 1:4,000, for the IgA and IgM tests or 1:10,000 for IgG test in a PBS-T/BSA buffer [phosphate-buffered saline containing 0.1% Tween (PBS-T) and 0.25% bovine serum albumin (BSA)]. Secondary antibodies (anti-human peroxidase-conjugated antibodies) for IgA, IgM, and IgG (Sigma, St. Louis, MO, USA) were used diluted 1:4,000 in PBS-T/BSA buffer. A microplate reader (Multiskan Sky Microplate Spectrophotometer, ThermoFisher, Waltham, MA, USA) was used to read the absorbance at 450 nm.

### HAI Assay

The hemagglutination inhibition (HAI) assay was performed as previously described by our group in Monteiro et al. (22). Briefly, we used the same volume (1:1, *v*/*v*) of chicken red blood cells (CRBCs) and IA H1N1 virus (concentration of 4 hemagglutination units per 50 μl in PBS) in 96-well round-bottom plates. Sera were serially diluted from 1:2 to 1:2,048, and then the diluted IA H1N1 virus was added. This mixture was incubated for at least 30 min at room temperature and CRBCs (1%) were added soon after. A new incubation for 30 min at room temperature with shaking was performed, and then the presence or absence of agglutination was analyzed. The HAI titer of the serum sample was determined as the inverse of the last dilution where cells were not agglutinated. A fourfold increase in the HAI titer post-vaccination, in relation to the values pre-vaccination, was considered significant (28).

### Determination of Naive and Effector CD8^+^ T Cells

**Figure 2:**
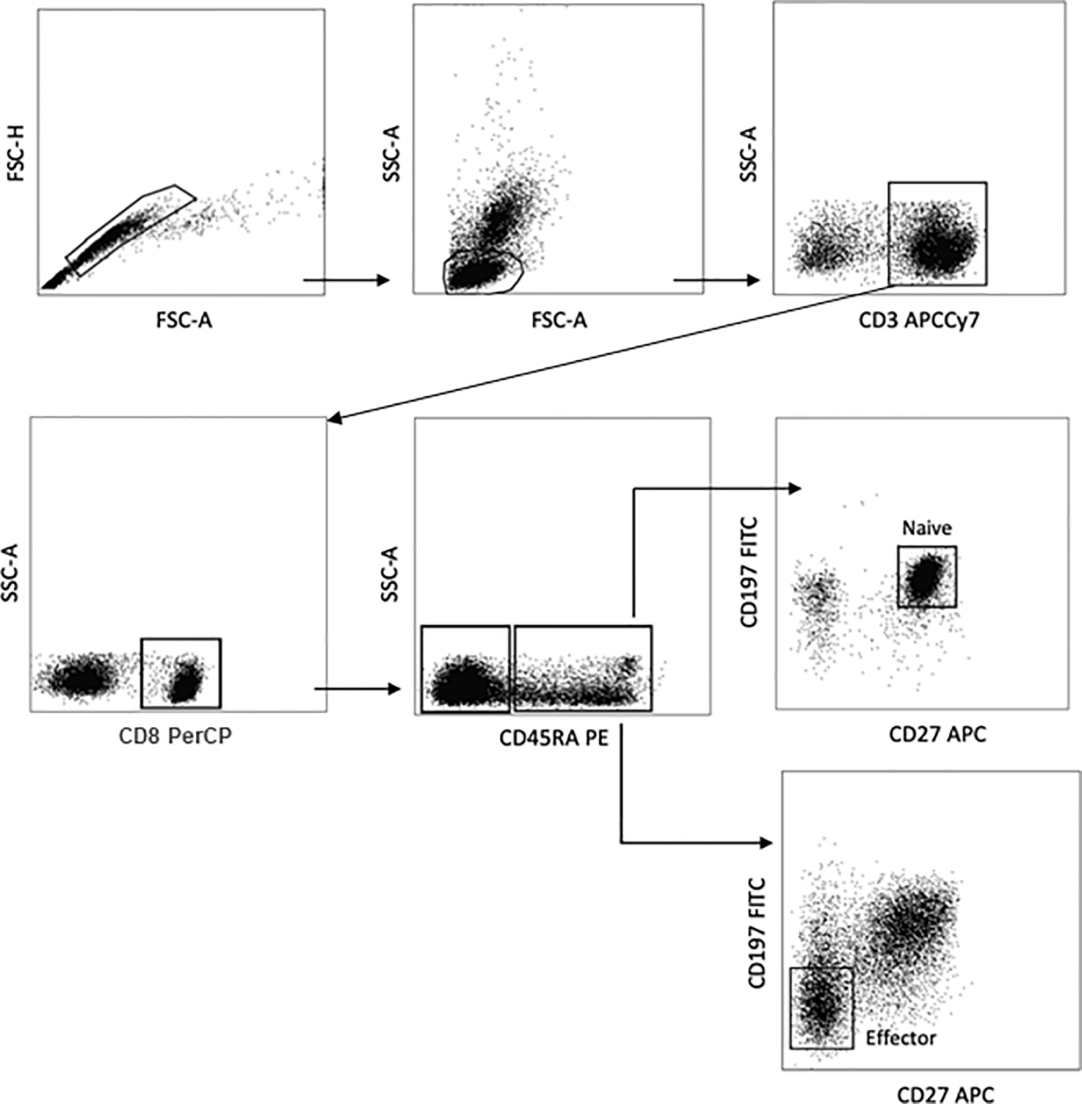
Flow cytometric analysis of cellular activation, naive and effector–memory CD8^+^ T cells. Representative dot plots of a flow cytometry panel used for the detection of naive and effector CD8^+^ T cells. Peripheral blood mononuclear cells (PBMCs) were stained with antibodies recognizing CD3, CD8, CD45RA, CD27, and CD197 and analyzed by flow cytometry, gating as indicated.

The immunophenotyping of CD8^+^ T cells, particularly in terms of naive and effector profiles, was performed as previously described by our group in Monteiro et al. (22). Briefly, 1 × 10^6^ cells were used for immunostaining with the monoclonal antibodies for FACS assays: anti-CD3 APCCy7 (clone SK7), anti-CD8 PerCP (clone clone SK1), anti-CD27 APC (clone O323), anti-CD45RA PE (clone HI100), and anti-CD197 FITC (CCR7, clone G043H7). The absolute numbers of naive and effector CD8^+^ T cells were determined as CD3^+^CD8^+^CD45RA^+^CD27^+^CD197^+^ and CD3^+^CD8^+^CD45RA^+^CD27^−^CD197^−^, respectively. The analysis strategies are shown in **Figure 2**. After immunostaining, 500,000 events were acquired on a FACSCanto II flow cytometer (BD Biosciences, San Jose, CA, USA) and then analyzed using the FlowJo software (version 10.2, BD Biosciences, San Carlos, CA, USA). In order to obtain robust and reliable results of the phenotypical characterization of CD8^+^ T cells, as also previously presented in Monteiro et al. (22), “an analysis strategy was carried out known as FMO (fluorescence minus one), which is widely used to identify more precisely where to select the cell population gates of interest when multiple fluorochromes are used in a given panel.”

### Determination of Pro- and Anti-Inflammatory Cytokines

Systemic pro-inflammatory (IL-6) and anti-inflammatory (IL-10) cytokine concentrations were determined in the serum samples using commercial ELISA kits (Invitrogen by Thermo Fisher Scientific, Vienna, Austria) following the manufacturer’s instructions. By using appropriate standard curves, which were obtained following instructions from manufacturers, it was possible to calculate each cytokine concentration. All correlation coefficients of the standard curves reached the range of 0.95–0.99, whereas the intra-assay and inter-assay coefficients of variance were 3%–7% and 7%–10%, respectively.

### Statistical Analysis

The data obtained were analyzed against the normality hypothesis using the Kolmogorov–Smirnov test, followed by the analysis of homogeneity of variance analyzed using the Levene test.

The anthropometric data and physical activity levels showed normal distribution and are presented as the mean and standard deviation. Based on these observations, unpaired Student’s *t*-test was used to determine significant differences in the data between the two volunteer groups.

In relation to the data for antibody responses to both IVV and CMV, and the cytokine levels, a deviation from normality (non-parametric variables) was found. Thus, these data were shown as median and interquartile ranges, and the Kruskal–Wallis test with the Müller–Dunn *post-hoc* test was used to determine whether the differences between these parameters in the older adult groups were significant. In addition, Spearman’s rank correlation coefficient analysis was applied to verify the significant correlations between the antibody responses to IVV and CMV and between the antibody responses and the cytokine levels.

The significance level was set to 5% (*p* < 0.05). All statistical analyses were performed using GraphPad Prism 8.1.2 software.

## Results

**Table 1** presents the results of the physical and anthropometric characteristics of the older adult NP and CET groups. As expected in a comparison between physically trained and untrained subjects, the NP group presented higher body weight, BMI, and total body fat, as well as lower fat-free mass, than did the CET group. The results of the IPAQ showed that the NP group presented lower times of physical activity in the week and higher sitting times than did the CET group. Moreover, **Table 1** also presents the percentage of volunteers in both groups who were responders or not to IVV (NP and CET groups), evaluated by the HAI assay.

**Table 1:** Physical and anthropometric characteristics of the older adult groups of non-practitioners (NP) and combined exercise training (CET) practitioners.

Characteristics	Volunteers (*n* = 80)		*p*-value
	NP (*n* = 31)	CET (*n* = 49)	
Age (years)	74.1 ± 6.4	71.7 ± 5.8	>0.05
Height (m)	1.57 ± 0.08	1.56 ± 0.09	>0.05
Weight (kg)	70.3 ± 11.2^a^	62.3 ± 10.4	0.004
Body mass index (kg/m^2^)	28.1 ± 3.9^a^	25.4 ± 3.7	0.002
Total body fat (%)	39.3 ± 9.6^a^	35.4 ± 7.5	0.04
Fat-free mass (%)	60.3 ± 9.3^a^	64.7 ± 7.6	0.02
Skeletal muscle mass (kg)	19.7 ± 4.5	20.1 ± 3.7	>0.05
IPAQ			
Physical activity (min/week)	438 ± 230^a^	708 ± 158	<0.0001
Sitting (min/week)	1,720 ± 565^a^	1,379 ± 497	0.02
Influenza vaccination responders, *n* (%)	32.2 (10/31)	32.6 (16/49)	>0.05
Influenza vaccination non-responders, *n* (%)	67.8 (21/31)	67.4 (33/49)	>0.05

IPAQ, International Physical Activity Questionnaire.

a Significant difference in relation to the values found in the CET group.

**Figure 3:**
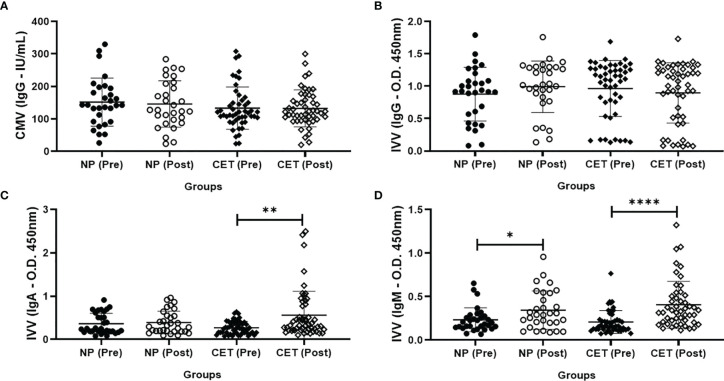
Serum concentrations (mean ± SD, in international units per milliliter) of the specific IgG antibody for cytomegalovirus (CMV) **(A)** and specific IgG **(B)**, IgA **(C)**, and IgM **(D)** antibodies for influenza virus vaccination (IVV; OD = 450 nm) in older adult non-practitioners (*NP*) and practitioners of combined exercise training (*CET*) both before (*Pre*) and 30 days after (*Post*) vaccination. *p < 0.05, **p < 0.01, **** p < 0.0001.

The specific IgG serum levels for CMV (**Figure 3A**) and IVV (**Figure 3B**) were unchanged between pre- and post-vaccination in both volunteer groups. In relation to the specific IgA serum levels for IVV (**Figure 3C**), the CET group showed higher concentrations post-vaccination than pre-vaccination (more than 2.5-fold, *p* < 0.01). Concerning the specific IgM serum levels for IVV (**Figure 3D**), both volunteer groups showed higher concentrations post-vaccination than pre-vaccination [1.5-fold in the NP group (*p* < 0.05) and 2.0-fold in the CET group (*p* < 0.001)].

**Figure 4:**
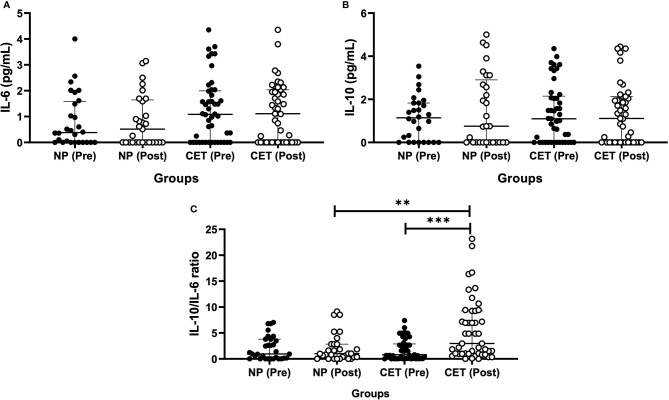
Serum concentrations (median and interquartile range) of IL-6 **(A)** and IL-10 **(B)** and the IL-10/IL-6 ratio **(C)** in older adult non-practitioners (*NP*) and practitioners of combined exercise training (*CET*) both before (*Pre*) and 30 days after (*Post*) vaccination. **p < 0.01, ***p < 0.001.

**Figure 4** shows the results obtained in the assessment of serum cytokines. For the serum levels of IL-6 (**Figure 4A**) and IL-10 (**Figure 4B**), no differences were found between the values pre- and post-vaccination in both volunteer groups. However, in the analysis of the IL-10/IL-6 ratio (**Figure 4C**), higher values were found in the CET group post-vaccination than pre-vaccination (*p* < 0.05).

**Figure 5:**
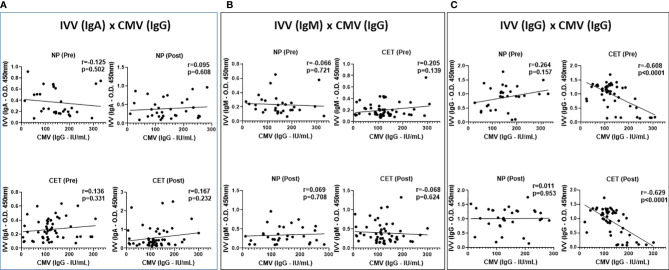
Spearman’s rank correlation coefficient analysis used to identify the correlation between the serum antibody levels for cytomegalovirus (CMV) and specific IgA **(A)**, IgM **(B)**, and IgG **(C)** for influenza virus vaccination (IVV) in non-practitioners (NP) and practitioners of combined exercise training (CET) at both pre- and post-vaccination periods. *p < 0.05.

**Figure 5** shows the correlation analysis between the serum antibody levels for CMV and IgA (A), IgM (B), and IgG (C), before and after the IVV, in the volunteer groups (NP and CET). As can be observed, no correlations were found between CMV and the IgA or IgM levels. However, whereas the NP group did not show any correlation between CMV and the IgG levels, the CET group showed a significant negative correlation both pre- and post-vaccination.

**Figure 6:**
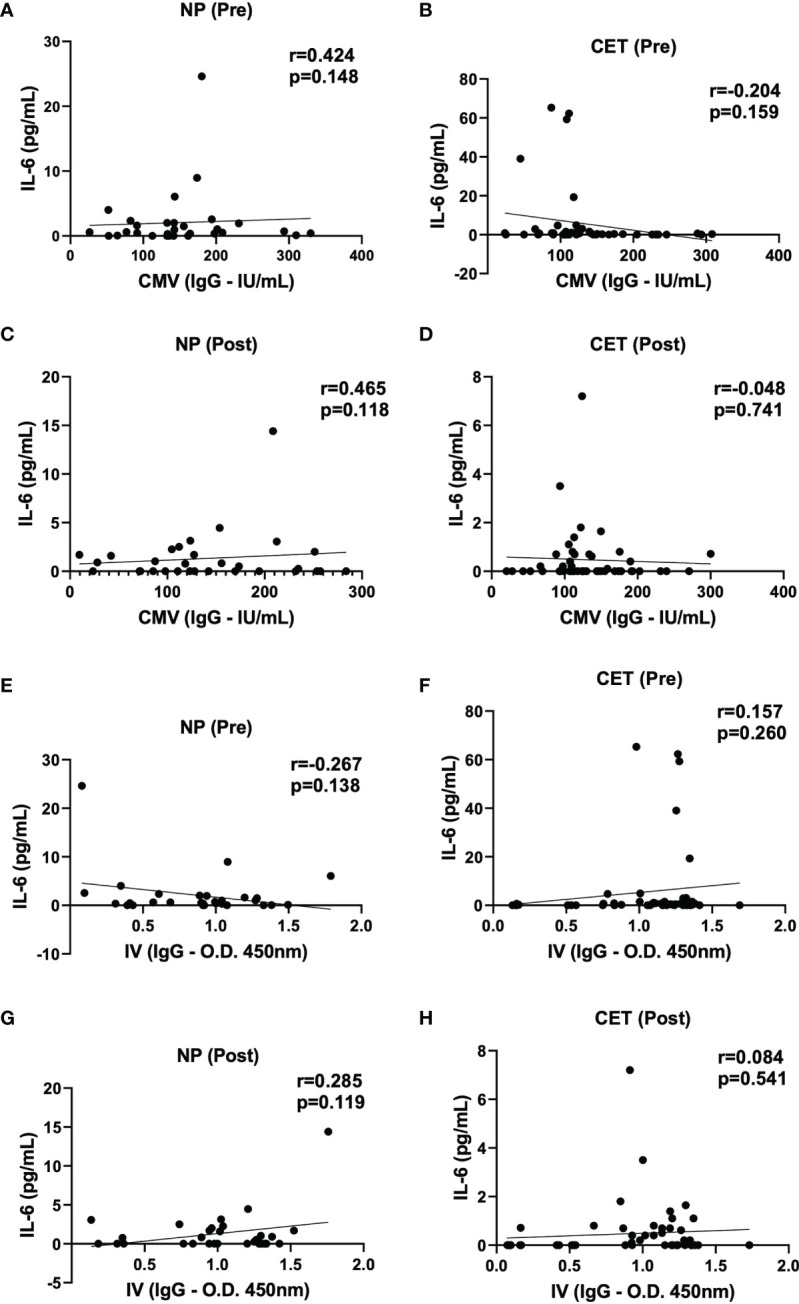
Spearman’s rank correlation coefficient analysis used to identify the correlation between the serum levels of IL-6 and specific IgG for cytomegalovirus (CMV) in non-practitioners (NP) and practitioners of combined exercise training (CET), pre-vaccination [NP **(A)** and CET **(B)**] and post-vaccination [NP **(C)** and CET **(D)**], and between IL-6 and specific IgG for influenza virus vaccination (IVV), pre-vaccination [NP **(E)** and CET **(F)**] and post-vaccination [NP **(G)** and CET **(H)**]. *p* < 0.05.

**Figure 6** shows the correlation analysis between the serum levels of IL-6 and specific IgG for CMV or IVV in the NP and CET groups pre- and post-vaccination. The results of this analysis showed that no significant correlations were found between the serum levels of IL-6 and specific antibody response to CMV or IVV.

**Figure 7:**
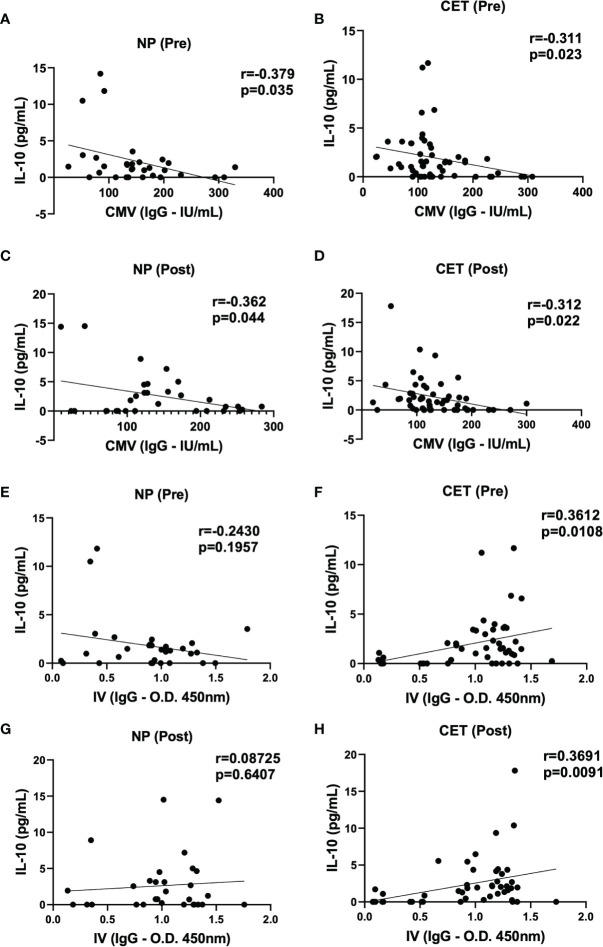
Spearman’s rank correlation coefficient analysis used to identify the correlation between the serum levels of IL-10 and specific IgG for cytomegalovirus (CMV) in non-practitioners (NP) and practitioners of combined exercise training (CET), pre-vaccination [NP **(A)** and CET **(B)**] and post-vaccination [NP **(C)** and CET **(D)**], and between IL-10 and specific IgG for influenza virus vaccination (IVV), pre-vaccination [NP **(E)** and CET **(F)**] and post-vaccination [NP **(G)** and CET **(H)**]. *p* < 0.05.

**Figure 7** shows the correlation analysis between the serum levels of IL-10 and specific IgG for CMV or IVV in the NP and CET groups pre- and post-vaccination. In this analysis between IL-10 and the specific antibody response to CMV, significant negative correlations were found in all groups at both pre- and post-vaccination periods (**Figures 7A–D**). Interestingly, in the correlation analysis between IL-10 and the specific antibody response to IVV, significant positive correlations were observed only in the CET group pre- and post-vaccination (**Figures 7E–H**).

Based on the observation that some participants from both groups were responders or non-responders to IVV, we carried out an analysis separating these volunteers into subgroups: “responders” and “non-responders.”

**Figure 8:**
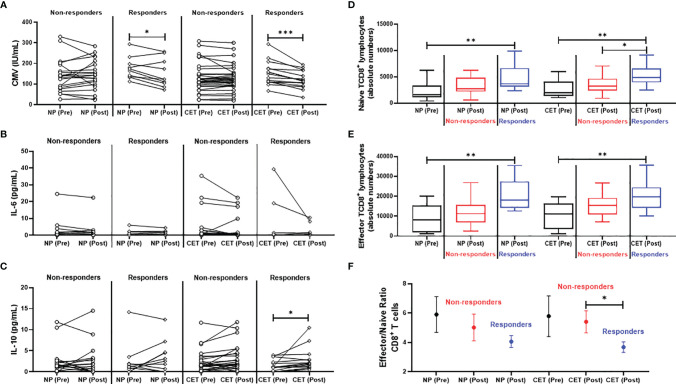
Comparison between the responders and non-responders of the non-practitioners (NP) and practitioners of combined exercise training (CET) groups to the influenza virus vaccination (IVV) for specific IgG for cytomegalovirus (CMV) **(A)** and levels of IL-6 **(B)** and IL-10 **(C)**, as well as naive **(D)**, effector **(E)**, and the effector/naive CD8^+^ T cell ratio pre- and post-vaccination. In **(D–F)**, the results of the non-responders are presented in *red*, whereas those of the responder are presented in *blue*. *p < 0.05, **p < 0.01, ***p < 0.001.

As shown in **Figure 8A**, older adult responders to IVV from both groups presented lower levels of specific IgG for CMV post-vaccination than pre-vaccination (NP: *p* < 0.05; CET: *p* < 0.001). **Figure 8B** shows that the IL-6 levels were unchanged between the subgroups of responders and non-responders at both pre- and post-vaccination. However, in **Figure 8C**, it is shown that the responder subgroup of the CET group presented a significant increase of the IL-10 levels post-vaccination compared to those pre-vaccination (*p* < 0.05). In addition, **Figures 8D, E** show that, whereas the responder subgroups of the NP and CET groups presented higher absolute numbers of naive and effector CD8^+^ T cells post-vaccination than pre-vaccination (*p* < 0.01), only the responder subgroup of the CET group presented increased absolute numbers of naive CD8^+^ T cells when compared to the non-responder subgroup of the CET group post-vaccination (*p* < 0.05). Finally, as shown in **Figure 8F**, a significant reduction in the effector-to-naive CD8^+^ T cell ratio was presented by the responder subgroup compared to the non-responder subgroup of the same CET group post-vaccination (*p* < 0.05).

## Discussion

In this study, we were able to demonstrate that, in agreement with the literature, older adult practitioners of the CET program had better specific antibody response (IgA and IgM) to IVV and higher IL-10/IL-6 ratios. Interestingly, both specific IgG for the vaccine and CMV, as well as the levels of IL-6 and IL-10, were unchanged in the time points evaluated here. However, from the HAI assay, it was found that similar percentages of volunteers in both groups were responders to IVV. While reductions in the levels of specific IgG for CMV and increases in naive and effector CD8^+^ T cells were found in both responder subgroups post-vaccination, only the responders of the CET group showed reductions in the ratio of effector to naive CD8^+^ T cells and increases in the IL-10 levels post-vaccination. In addition, significant correlations were observed. In this respect, negative correlations between the specific IgG levels for the vaccine and CMV were found in the CET group and between IL-10 and specific IgG levels for CMV in all volunteer groups pre- and post-vaccination. In addition, a positive correlation between IL-10 and specific IgG for IVV was observed pre- and post-vaccination. Lastly, the levels of IL-6 did not show correlations with the other parameters evaluated.

Since the term inflammaging was coined, several studies have been developed to improve knowledge on the impact of the inflammatory status on the aging process. Regarding the current data, the inflammaging phenomenon is characterized by higher systemic and chronic levels of tumor necrosis factor alpha (TNF-α) and IL-6, both pro-inflammatory cytokines, in association with lower levels of IL-10, a classical anti-inflammatory cytokine (3). Some reports pointed out that this imbalance between the pro- and anti-inflammatory cytokines in older adults can be considered one of the main causes of the increased risk of developing many diseases, such as sarcopenia, atherosclerosis, and Alzheimer’s, besides interfering with the immunological response and vaccination responses (7, 29). Although the mechanisms that trigger the inflammaging phenomenon are not fully understood, it is widely accepted that obesity and CMV infection are two factors involved not only in the triggering but also in the maintenance of this harmful age-related phenomenon.

A relevant proof for CMV infection being a remarkable piece in the puzzle of inflammaging is that CMV is able to promote the activation and translocation of NF-κB from the cytoplasm to the nucleus, inducing an increase of TNF-α and IL-6 production, leading to an upregulation of the pro-inflammatory response (13, 30–32). Based on the literature, CMV has the capacity to maintain a long-standing latency state within the host. It should be emphasized that the prevalence of CMV infection is higher and that its infection rates increase with age, reaching up to 95% in older adults (33–35).

Despite CMV being able to induce IL-6 production, as cited above, in this study, we did not observe any correlation between the serum levels of specific IgG for CMV and IL-6 in all volunteer groups in the time points evaluated. On the contrary, the correlation analysis between the serum levels of specific IgG for CMV and IL-10 showed negative correlations in the two groups, both pre- and post-vaccination. Particularly, this last finding can reinforce our former description that CMV infection occurs and is maintained in a higher pro-inflammatory and a reduced anti-inflammatory environment (21) since the higher levels of CMV antibodies were found in the participants with lower circulating IL-10 levels.

Another point that needs highlighting is that there is no consensus on the impact of CMV infection on IVV. In fact, some studies have demonstrated that CMV seropositivity is negatively associated with specific antibody response to IVV, while other studies did not show any association between CMV seropositivity and IVV, mainly in the older adults (12, 36–39). Of utmost importance is that it was reported that older adults with higher CMV seropositivity presented reduced responses of CD4^+^ T cells and, more prominently, of CD8^+^ T cells for influenza proteins, which demonstrates that CMV infection could influence the immune response against the influenza virus (13). Furthermore, also worth citing is that, in contrast to that observed in older adults, young adults with CMV infection showed an enhancement of the immune response to IVV (7). In our study, we found that the serum levels of specific IgG for CMV and IVV remained unchanged at both pre- and post-vaccination in all volunteer groups. Although our group has demonstrated that older adults who exercise showed improvement in specific IgG for IVV (4), more recently, we also found that a group of older adults who practice exercise training did not show alterations in the specific IgG levels for IVV (22). As mentioned in this former study (22), seasonal IVV, unfortunately, can negatively influence the immune response (40) and no improvement in the specific IgG levels for the vaccine can be found (22).

Among several aspects that could explain the lack of immune response observed after IVV, the vaccine dose used has been reported to have a singular influence on eliciting a robust immune response in the older population. In this respect, there are studies, both original and systematic meta-analysis, in the scientific literature highlighting that, among older people, the high-dose influenza vaccine (60 μg of hemagglutinin per virus strain) is more immunogenic, well tolerated, and can provide better protection against influenza infections than does the standard-dose vaccine (15 μg of hemagglutinin per virus strain) (41–43). Based on these data, we can suggest that the unaltered specific IgG levels 30 days after IVV could be attributed, at least in part, to the standard-dose vaccine that was administrated in the participants in the present study.

Although our group has demonstrated the antibody responses to IVV, by comparing the data obtained pre- and post-vaccination, in an older population through the optical density (OD) values from the ELISA, we also evaluated the antibody titers with the HAI assay in order to better understand the immune response elicited by IVV. In agreement with the literature, analysis of the antibody titers is a well-accepted tool for the evaluation of protection from the IVV (28, 44). Thus, by using the HAI assay, we were able to verify that similar percentages of older adults in both volunteer groups were responders to the IVV. Interestingly, these responders showed a significant reduction of the levels of specific IgG for CMV post-vaccination. It is worth clarifying that all the volunteers enrolled in this study were CMV-seropositive, and these observations can add more pieces to this puzzle. Moreover, when we evaluated the results from the correlation analysis, whereas the NP group did not present any correlation, the CET group showed a significant negative correlation between the values of specific IgG for CMV and IVV. Considering these findings, we can putatively suggest that the regular practice of combined exercise training could be useful not only to improve but also to drive the specific antibody response to CMV or IVV in older people.

It is well known that the regular practice of exercise training is a non-pharmacological intervention that benefits the immunological system due to the production, differentiation, maturation, and activation of leukocytes until their actions in order to guarantee protection against several immunological challenges, such as vaccination in older adults (45, 46). Indeed, this prominent capacity of exercise training to enhance vaccine efficacy is more pronounced in situations where the vaccination shows low immunogenicity, as in immunosenescence (45). In this sense, our group has demonstrated that CET can significantly improve not only the systemic immune responses, both cellular and humoral, but also the immune responses in the mucosal airways against IVV in older adults (22, 23). Corroborating these pieces of information, we presented in this study that the volunteer group that practiced the CET program showed an enhancement of the serum levels of specific IgA and IgM post-vaccination in comparison to the baseline values. It is worth mentioning that the increase of the serum levels of specific IgM post-vaccination in comparison to those at baseline in the NP group can be attributed to the fact that the volunteers in this group were classified as active older adults. This last observation can also reinforce the data that an active lifestyle has the ability to mitigate the immunosenescence and, consequently, to preserve the immunological response during aging.

Even though an improvement in the serum levels of specific IgG for IVV was expected, particularly in the CET group, the observation of differences in the correlation analysis between the two volunteer groups can be corroborated by some data in the literature (47). In this respect, it has been postulated that there is an inverse association between vaccine efficacy and CMV serostatus, as observed in this study exclusively in the CET group. Furthermore, it is paramount to point out that it has been reported that the control of CMV infection associated with a significant reduction of the viral burden imposed on the immune system through regular practice of exercise training was able to improve the vaccine effectiveness. Also importantly, in accordance with the literature, the remarkable effect of exercise training in this context cannot be observed in individuals who presented good control of CMV infection (47).

Despite the real effect of exercise training in this context not being fully determined, some studies postulated that the benefits of this intervention can be attributed to its ability to improve a T-cell turnover, in which T cells presenting a senescence phenotype are induced to die by apoptosis and that the “space” will be filled by new functional T cells (22, 47–50). In consequence, the renewal of a pool of T cells from a senescence aspect to a functional profile can restrain or decrease the CMV infection, which can also reduce the antigenic stimulus and the number of “older” CMV-specific T cells, leading to the control of CMV reactivation (47, 51), since around 25% of the CD8^+^ T cells can show specificity for a unique CMV-immunodominant epitope in CMV-seropositive older adults (29). As is well known, immunological control of CMV infection requires permanent immune surveillance, particularly with an increase of up to 10%–30% of CMV-specific CD8^+^ T cells in the periphery, which remains to elevate over time. As already mentioned, latent CMV infection frequently shows a remarkable decrease in naive T cells, particularly in CD8^+^ than in CD4^+^ T cells, when compared to that without CMV infection. In contrast to these reductions in naive CD8^+^ T cells, CMV-seropositive individuals present an elevation in the percentages of effector–memory CD8^+^ T cells, which can lead to an impairment of the immune response [12, 15–19]. In this regard, it was reported that CMV-specific CD8^+^ T cells presented an advanced differentiated phenotype, which frequently is characterized by the expressions of CD45RA and CD57 in association with the lack of CD27, CD28, and CCR7. In contrast, naive CD8^+^ T cells present the expressions of CD45RA, CD27, CD28, and CCR7 (28, 52, 53). Based on these data, the modulation of naive and effector CD8^+^ T cells is useful to achieve a continuous and active immune response not only to CMV infection but also to other immunological challenges, such as respiratory viruses and/or vaccines (28, 53). Corroborating this suggestion, we observed that the subgroup of older adult responders to IVV showed an elevation of both naive and effector CD8^+^ T cells; in particular, the older adults who practice physical exercise also showed significant decreases in the ratio of effector to naive CD8^+^ T cells. This remarkable finding agrees with the literature, which showed that combined exercise training drives to increase the number of CD28^+^CD8^+^ T cells in the periphery (54, 55) in older adults, and allows us to suggest that the regular practice of CET is able to improve the number of naive CD8^+^ T cells in relation to effector CD8^+^ T cells, which leads the immune response to maintain the capacity to respond to new immunological challenges.

As appealing as this proposal is, it is worth highlighting that another widely accepted and positive effect associated with the regular practice of exercise training is its property to generate a systemic anti-inflammatory status (56). It has been reported that exercise training can upregulate the production of anti-inflammatory cytokines, such as IL-10, in association with the downregulation of pro-inflammatory cytokines, such as IL-6 and TNF-α (57, 58). As reported by Jankord et al. (57), the regulation of systemic inflammatory status, mainly by the action of IL-10, is related to a protective environment that may be able to minimize the harmful effect of aging.

As formerly described, the inflammaging phenomenon is characterized by an imbalance of pro- and anti-inflammatory cytokines. In this context, some studies demonstrated that circulating IL-10 levels are decreased during aging (59), which can allow the increase of pro-inflammatory cytokines, especially IL-6. Therefore, the capacity of exercise training to enhance the levels of IL-10 is helpful in controlling the inflammation process since this cytokine presents a suppressive effect on the secretion of many pro-inflammatory cytokines (60). Although our results did not show differences in the serum levels of IL-6 and IL-10 between the two volunteer groups, which can be attributed not only to the fact that all volunteers had an active lifestyle but also to the observation that moderate exercise training promotes short time fluctuations in some cytokine levels (61), the IL-10/IL-6 ratio showed a significant increase in the CET group post-vaccination. Currently, analysis of the pro- to anti-inflammatory cytokine ratio is useful in providing a better view of the real context of inflammatory status (62–64). Thus, our observation of the CET group increasing the IL-10/IL-6 ratio allows us to suggest that an improvement of the systemic inflammatory status was achieved in this group, which could positively influence better specific antibody responses (IgA and IgM) to IVV, as observed here.

Corroborating its prominent anti-inflammatory property, the serum levels of IL-10 showed a significant negative correlation with specific IgG for CMV in all volunteer groups both pre- and post-vaccination. Based on this result, we can suggest that older adults who maintain regulation of inflammatory status, particularly through IL-10 activity, are able to better control CMV infection. Additionally, we observed that, in the CET group, the IL-10 levels were positively correlated with the specific IgG levels for the antigens presenting in the influenza virus vaccine. Furthermore, the results observed from the division of the CET group into subgroups of responders or non-responders to IVV allow us to reinforce this suggestion since an increase in the IL-10 levels was evidenced post-vaccination only in the responder subgroup, and this anti-inflammatory regulation could impact improvement of the immune response to the vaccine. In fact, this observation is very important since it has been reported that alterations in the levels of IL-10 can impair the immune response to IVV in older people (65, 66).

Taken together, the results obtained in this study showed, for the first time, that CMV-seropositive older adults, especially those who physically train, present an improved response to IVV in association with an anti-inflammatory status and enhancement of naive CD8^+^ T cells.

## Data Availability Statement

The raw data supporting the conclusions of this article will be made available by the authors, without undue reservation.

## Ethics Statement

The studies involving human participants were reviewed and approved by the Ethics Committee of the Federal University of São Paulo (approval no. 3.623.247). The patients/participants provided written informed consent to participate in this study.

## Author Contributions

EF, JS, and AB conceived the study, analyzed the data, and, together with ED, DO, MV, RV, and CF, wrote the initial, final, and revised drafts of the manuscript. MR, CS, and GA participated in the planning and development of this study. LT, JSA, FM, AS, and JA participated in the laboratory analysis. AB and ED supervised and acquired funding. All authors contributed to the article and approved the submitted version.

## Funding

Funding was granted by Fundação de Amparo à Pesquisa do Estado de São Paulo (FAPESP): grant nos. 2012/15165-2 (to RV), 2016/20045-7 and 2020/06409-1 (to ED), and 2010/50025-1 and 2019/14115-0 (to AB).

## Conflict of Interest

The authors declare that the research was conducted in the absence of any commercial or financial relationships that could be construed as a potential conflict of interest.

## Publisher’s Note

All claims expressed in this article are solely those of the authors and do not necessarily represent those of their affiliated organizations, or those of the publisher, the editors and the reviewers. Any product that may be evaluated in this article, or claim that may be made by its manufacturer, is not guaranteed or endorsed by the publisher.
